# Modified hemispherectomy for infantile hemiparesis and epilepsy

**DOI:** 10.1515/tnsci-2020-0145

**Published:** 2020-10-20

**Authors:** Yu-Hui Li, Dong-Sheng Li, Mei-Qing Wang, Kai Zhao, Bu-Lang Gao

**Affiliations:** Department of Neurosurgery, Shijiazhuang People’s Hospital, Xian Jiaotong University, 365 South Jianhua Street, Shijiazhuang, Hebei Province 050030, China

**Keywords:** infantile hemiparesis, refractory epilepsy, hemispherectomy, medical imaging, complications

## Abstract

**Objective:**

To investigate the effect and medical imaging of modified hemispherectomy on patients with infantile hemiparesis and medically refractory epilepsy.

**Patients and methods:**

Forty-three patients with infantile hemiparesis and refractory epilepsy who underwent hemispherectomy were enrolled. The treatment effect and medical imaging were analyzed.

**Results:**

Anatomical hemispherectomy was successfully performed in all patients (100%). In all patients, the muscular tension decreased and the contracted limbs relaxed. In the pathological examination of the resected brain tissue, secondary cicatricial gyri with concomitant cortical dysplasia was present in 36 cases and polycerebellar gyrus malformation and porencephalia in the other 7 cases. Followed up for 7–15 years (mean 11.3), all patients were alive without a long-term sequela. Epilepsy was satisfactorily controlled, with complete seizure relief in 39 cases (91%) classified as Engel I and basic control in the other 4 (9%) defined as Engel II. The posthemispherectomy medical imaging demonstrated that the intracranial space on the operative side shrank, and the healthy cerebral hemisphere shifted markedly toward the hemispherectomy side, with expanded lateral ventricle on the healthy side and thickened skull and enlarged frontal sinus on the operative side. After 4–5 years, the intracranial space on the operative side disappeared in 75% of the patients, demonstrating enlarged cerebral peduncle on the healthy side.

**Conclusion:**

Further modified hemispherectomy in patients with infantile hemiparesis and medically refractory epilepsy demonstrated markedly ameliorated effects on epilepsy control and the prevention of superficial cerebral hemosiderosis in the long-term follow-up.

## 1 Introduction

As an established procedure of surgery, anatomical hemispherectomy has been used for treating disabling, medically intractable epilepsy resulting from diffuse hemispheric diseases [1–3]. Since the introduction of this surgical technique as a therapeutic approach for epilepsy in 1938 [4], various modifications have been reported to ameliorate the outcome and minimize procedure-related complications [4,6–8]. The rate of seizure freedom following hemispherectomy has been reported to be 52–89%, with a link in improved seizure control and cognitive motor outcomes [3,9–11]. The postoperative mortality rate of hemispherectomy in recent decades has been shown to range from 0 to 1% [3] or 6.6% [12]. Delayed deadly intracranial complications may occur after many years without epilepsy, with an incidence of approximately 30% complications [1,7]. These delayed complications include hydrocephalus of normal pressure, development of a peculiar vascular membrane lining the operative cavity and the remaining ventricles, and superficial hemosiderosis of the brain and spinal cord [1,13,14]. These complications were thought to be caused by many events of tiny hemorrhage into the large subdural space created after the diseased hemisphere was removed, leading to superficial cerebral hemosiderosis and granular ependymitis [1,7]. Modified and improved techniques in hemispherectomy are associated with better clinical outcomes. In 1983, Adams revised the anatomic hemispherectomy by plugging the Monro foramen with muscle and plicating the convexal dura to the cerebral falx and the cerebellar tentorium at the middle cranial fossa, with the purpose of decreasing the subdural cavity, blocking communication of the subdural space with normal cerebrospinal fluid pathway, and finally preventing superficial cerebral hemosiderosis and other complications [15]. Peacock drained the hemispherectomy space for 3–5 days following the procedure before the placement of a subdural–peritoneal shunt to drain blood products known to induce the formation of subdural membrane and superficial cerebral hemosiderosis [16]. None of the patients developed superficial cerebral hemosiderosis in the studies by Adams [15] and Peacock et al. [16]. The method used by Adams [15] could block bloody fluid from entering the cerebrospinal fluid circulation; however, the piece of muscle used to plug the foramen of Monro might easily be displaced by alteration of body position or with circulation of the cerebrospinal fluid, thus losing its role in preventing bloody cerebrospinal fluid from entering the hemispherectomy cavity. To prevent the muscle piece from displacement, the procedure was modified by suturing the muscle plug to the cerebral falx and cerebellar tentorium and tightly sealing the space surrounding the muscle piece with biological glue. Moreover, when plicating and suturing the convexal dura to the cerebral falx and the cerebellar tentorium, the subdural space was made as small as possible, and the suture sites were glued tightly for complete seal to prevent bloody fluid in the epidural space from entering the subdural space on the hemispherectomy side. After hemispherectomy, the wound surface is very large and the oozing of blood will continue no matter how perfect hemostasis has been performed. Tight seal can prevent bloody cerebrospinal fluid from entering the subdural space, and the hemispherectomy space should be drained for 3–5 days following the procedure.

The volume of cerebral peduncle ipsilateral to the hemispherectomy can be used to predict postoperative hemiparesis, with a smaller ipsilateral peduncle volume being predictive of improved postoperative motor function on the contralateral side [17,18]. This is because smaller peduncle volumes indicate fewer motor fibers stemming from the ipsilateral hemisphere, and hemispherectomy to remove nonfunctional hemisphere of the brain leads to fewer postoperation deficits. A smaller cerebral peduncle on the ipsilateral side of hemispherectomy was potentially associated with nonworsened postoperative hemiparesis, which may indicate reorganization of corticospinal tract in childhood in the smaller ipsilateral cerebral peduncle and noncontribution of the removed cerebral hemisphere to the contralateral motor function [19]. In our modified anatomic hemispherectomy, the effect of the surgery and changes in the skull and brain peduncle after the surgery were unknown, and this study was consequently performed to investigate the effect of modified hemispherectomy on patients with infantile hemiparesis and concurrent medically intractable epilepsy and changes in the brain and skull on medical imaging.

## 2 Materials and methods

Between March 1995 and December 2003, patients with hemiplegia concomitant with medically refractory epilepsy who underwent hemispherectomy were enrolled. The inclusion criteria were patients with hemiplegia combined with intractable epilepsy, with imaging examination of computed tomography (CT) or magnetic resonance imaging (MRI).

All patients underwent electroencephalogram (EEG), imaging, and physical examination to confirm the disease location 2–5 days before the surgery. EEG demonstrated abnormalities in all cases, and the basilic rhythm showed slow-spike waves, with flat waves, spike waves, and polymorphic δ waves on the diseased side. CT or MRI before operation showed atrophy, softening lesions, and calcification of varying degrees on the diseased cerebral hemisphere, while the cerebral peduncle on the healthy hemisphere was thickened.

All patients underwent the modified hemispherectomy procedure in our hospital, and then, the residual cavity was managed with the modified methods. First, following hemispherectomy and removal of the diseased hemisphere, an appropriate slice of muscle was used to occlude the interventricular foramen of Monro and fixed onto the frontal and rear parts of the cerebral falx. Then biological glue was used to seal the residual space around the muscle plug. Second, the convexal dura mater on the hemispherectomy side was completely cut out, made into a piece of an appropriate size, and sutured onto the cerebellar tentorium for complete sealing of the cerebellar tentorial foramen before reinforcement with biological glue to seal the surrounding residual gap. After hemispherectomy, a drainage tube was placed to drain bloody cerebrospinal fluid for 3–5 days until the cerebrospinal fluid became clear. After hemispherectomy, pathological examination of the hemisphere was performed, and antiepileptic drugs were administered. Long-term follow-up was required, and EEG and medical imaging examination were also performed. Two years after the procedure, antiepileptic drugs were gradually reduced if no epilepsy occurred, and the process of drug reduction took over half a year.

The Children’s global assessment scale (CGAS) was used to evaluate the patients’ social, behavioral, and emotional functioning before surgery and at follow-up [20,21]. This scale ranges from 0 (very poor functioning) to 100 (very high functioning) and is believed to have adequate test–retest reliability and good discriminative and concurrent validity and reliability between different raters.


**Ethical statement:** The research related to human use has been complied with all the relevant national regulations, institutional policies and in accordance the tenets of the Helsinki Declaration, and has been approved by the authors’ institutional review board or equivalent committee.
**Informed consent:** Informed consent was obtained from all patients or their guardians for being included in the study.

## 3 Statistical analysis

The statistical analysis was performed with the SPSS version 21.0 (IBM, Chicago, IL, USA). The CGAS scores were presented as mean ± standard deviation and compared before surgery with those at follow-up using the paired *t* test. The significant *P* value was set at <0.05.

## 4 Results

Forty-three patients with hemiplegia combined with intractable epilepsy were enrolled, including 28 male and 15 female patients in the age range of 7–22 years (mean 13.6). Anatomical hemispherectomy was performed in 18 cases on the left and 25 on the right side. The hemispherectomy was successful in all patients (100%). All patients had infantile hemiplegia concomitant with medically refractory clonic epilepsy. The time of first onset of seizure ranged from 2 days following birth to 17 years (mean 6 years), with the seizure frequency ranging from 1/month to 10/daily. Physical examination revealed typical hemiplegia of limbs including left hemiplegia in 25 cases and right in 18, with the muscle strength significantly increased from II to III on the hemiplegia side concomitant with muscular atrophy, tendon contracture, and positive pathological sign. Thirty-seven patients had abnormal personality and behavior of impulse, aggression, and irritability. The CGAS before surgery ranged from 33 to 79 (mean 60.2 ± 11.7).

After hemispherectomy, fever occurred in 35 patients with a duration of 3–12 days. Lumbar drainage was performed for bloody cerebrospinal fluid, which showed no infection after laboratory test. The muscle strength on the hemiplegia side decreased in five cases and resumed after half a year. Compared to the muscle strength before surgery, the muscle strength in the other 38 patients did not deteriorate on the hemiplegia side but increased by more than one grade at follow-up. The muscular tension in all patients decreased and the contracted limbs relaxed. Pathological examination of the resected brain tissue showed secondary cicatricial gyri with concomitant cortical dysplasia in 36 cases and polycerebellar gyrus malformation and porencephalia in the other 7 cases. Mental disorders were apparently relieved in 37 patients with abnormal personality and behavior. Fundoscopy was performed in every patient, which showed the fundus artery to vein ratio of ≥2:3 and clear boundary of the optic disc.

Follow-up at 7–15 years (mean 11.3) demonstrated that all patients were alive without long-term complications or sequelae. Epilepsy was satisfactorily controlled, with complete seizure relief in 39 cases (91%) classified as Engel I and basic control in the other 4 (9%) defined as Engel II. In five patients, the limb movement function on the hemiplegia side decreased slightly but the elevated muscular tension decreased apparently when compared to that before surgery. After rehabilitation therapy, the limb muscle strength increased to that before surgery. Regarding the linguistic ability, one patient had aphasia which was resumed to that before surgery after treatment. The other patients all had more fluent language expression compared to that before surgery. At follow-up, the CGAS score ranged from 59 to 93 (mean 78.5 ± 9.4), which was significantly (*P* < 0.0001) better than that before surgery.

**Figure 1:**
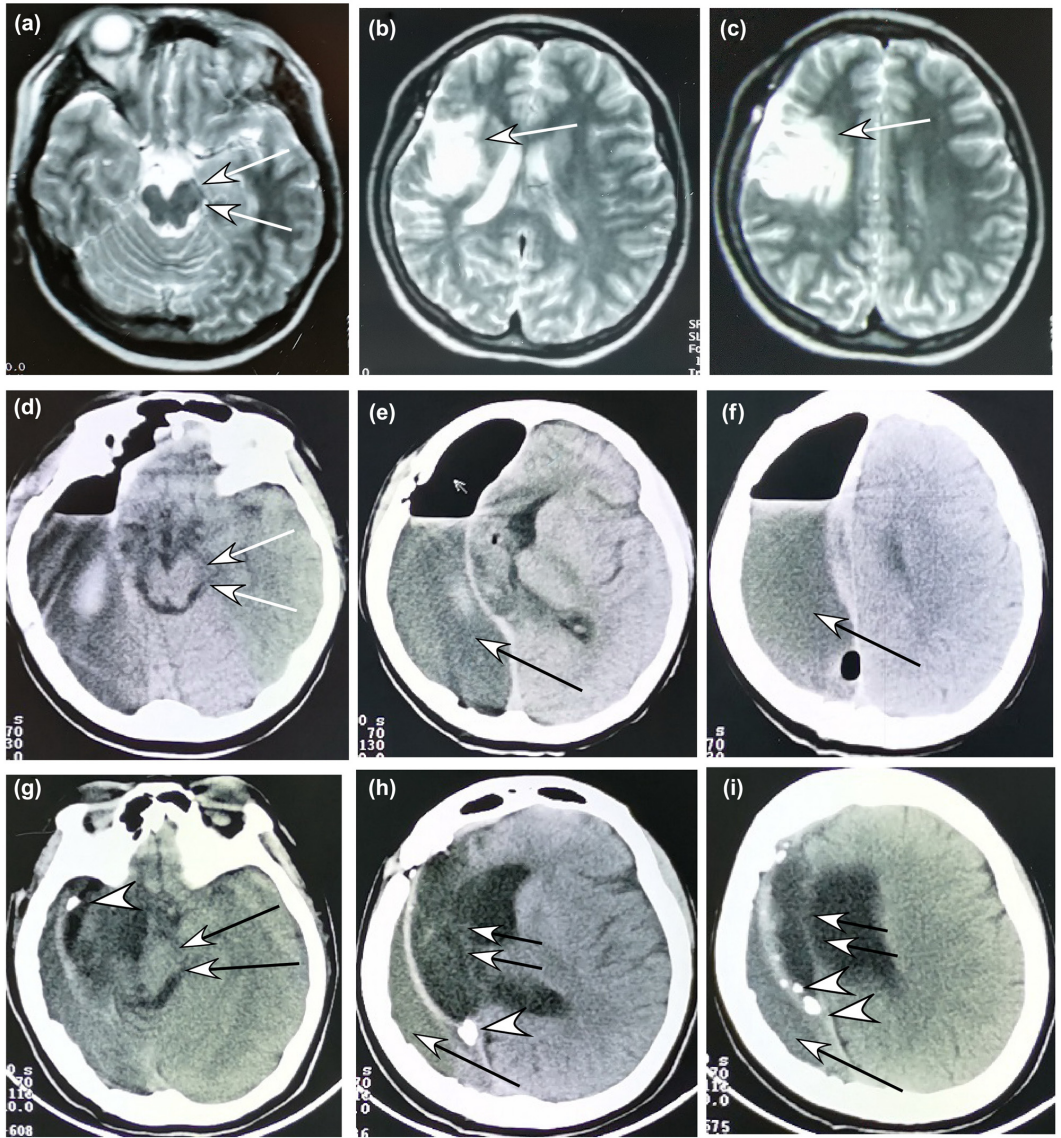
A 16-year-old girl had medically refractory epilepsy and underwent hemispherectomy. (a–c) Before the hemispherectomy, magnetic resonance imaging of the brain revealed compensatory enlargement of the left cerebral peduncle but atrophic right cerebral peduncle (arrow in A) and atrophic brain tissue (arrow in B and C). (d–f) Computed tomographic scan (CTS) at 7 days posthemispherectomy demonstrated enlarged cerebral peduncle (arrows in D) on the left side and a residual intracranial space (arrow in E and F) on the right side with accumulation of air and fluid. (g–i) CTS 3 years later showed enlarged cerebral peduncle on the left side (double arrows in G), right-shifted center line (double arrows in H and I), and shrank epidural space (bigger arrow in H and I) under the skull. Calcification (arrow head in G–I) occurred on the dura mater bordering the shrank epidural space and the left hemisphere, and this calcified dura mater gave a support to the left hemisphere.

**Figure 2:**
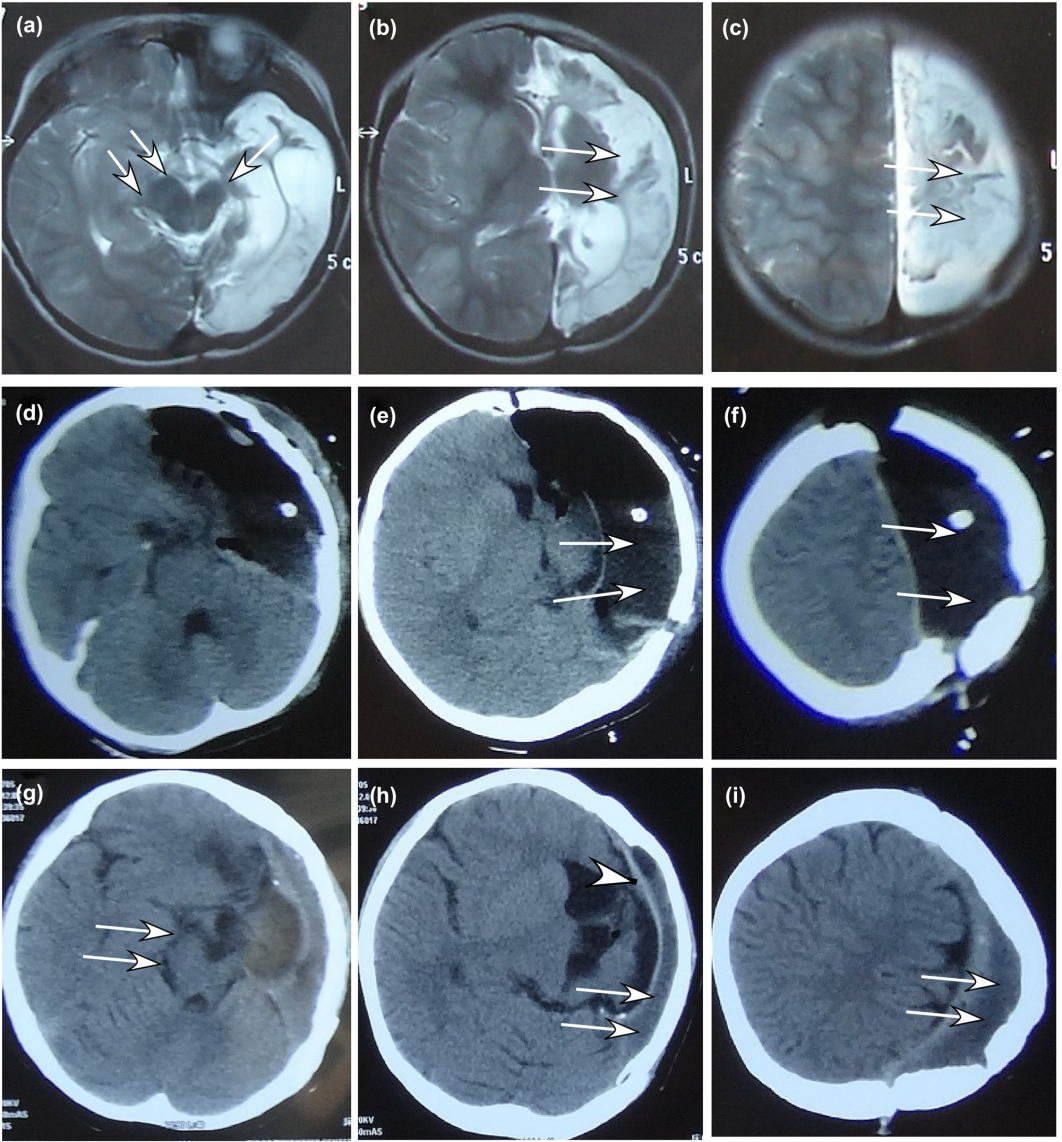
An 8-year-old girl with infantile hemiparesis and medically intractable epilepsy had undergone anatomical hemispherectomy. (a–c) Before the hemispherectomy, magnetic resonance imaging of the brain revealed compensatory enlargement (double arrows in A) of the right cerebral peduncle, atrophic left cerebral peduncle (single arrow in A), and atrophic brain tissue (double arrows in B and C). (d–f) CTS at 10 days posthemispherectomy demonstrated left-shifted midline structures and the residual intracranial space (double arrows in B and C) with accumulated air and fluid. (g–i) CTS 3 years later revealed enlarged cerebral peduncle on the right (double arrows in G), further left-shifted midline structures and shrank epidural space (double arrows in H and I) under the thickened skull. The arrow head indicates the dura mater separating the residual epidural space and the brain tissue on the right.

**Figure 3:**
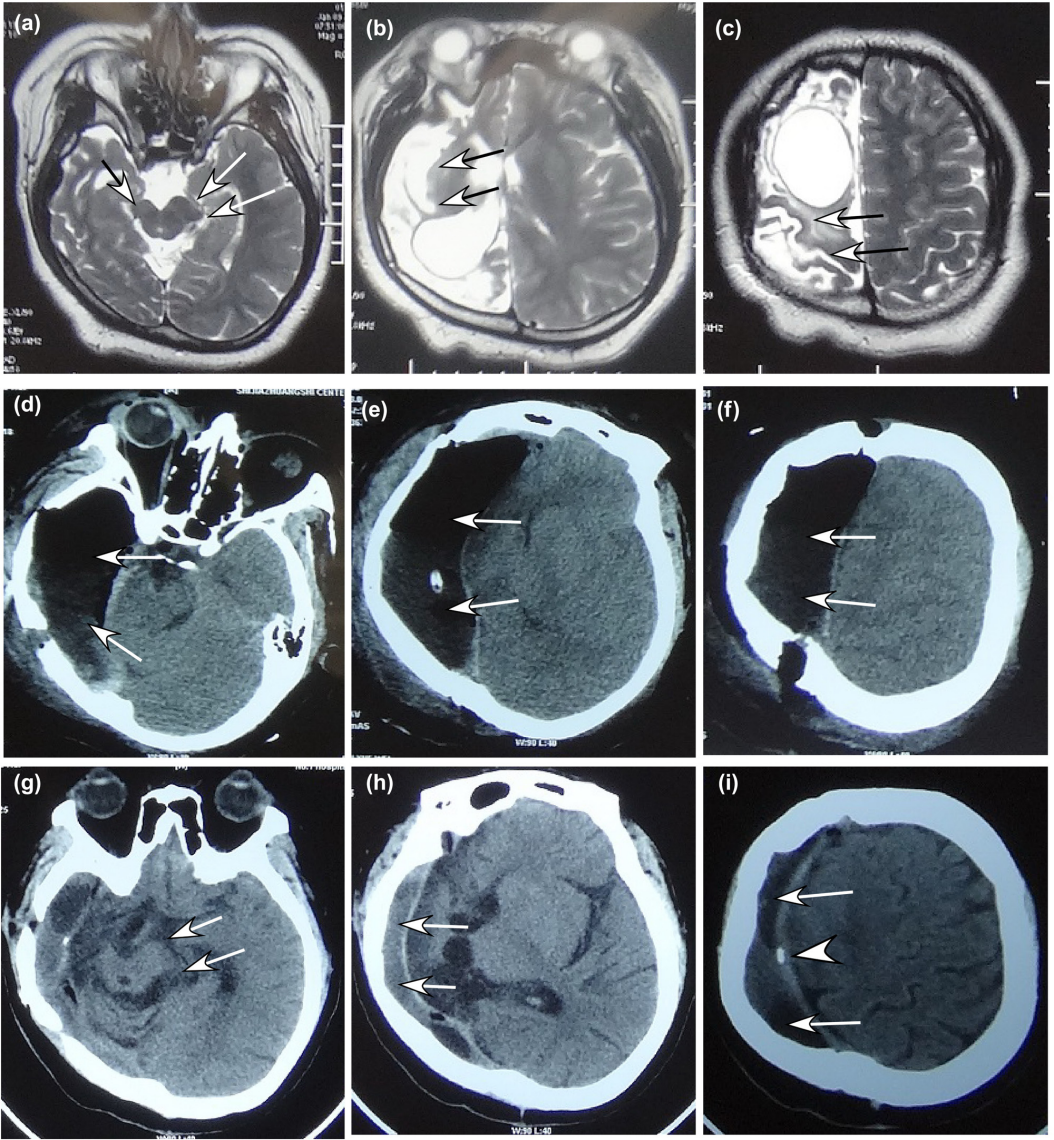
A 21-year-old girl had anatomical hemispherectomy because of cerebral hemorrhage at 1 month after birth and medically intractable epilepsy for 16 years. (a–c) Before the hemispherectomy, magnetic resonance imaging of the brain revealed compensatory enlargement (double arrows in A) of the left cerebral peduncle, atrophic right cerebral peduncle (single arrow in A), and atrophic brain tissue on the right (double arrows in B and C). (d–f) CTS at 8 days posthemispherectomy demonstrated right-shifted midline structures and the residual intracranial space (double arrows) with accumulated air and fluid. (g–i) CTS 3 years later revealed enlarged cerebral peduncle on the left (double arrows in G), further right-shifted midline structures and shrank epidural space (double arrows in H and I) under the thickened skull. The arrow head indicates calcification of the dura mater separating the residual epidural space and the brain tissue on the left.

The medical imaging posthemispherectomy (Figures 1–3) showed that the intracranial space on the operative side shrank and the healthy cerebral hemisphere shifted markedly toward the hemispherectomy side, with the expanded lateral ventricle on the healthy side and thickened skull and enlarged frontal sinus on the operative side. After 4–5 years, the intracranial space on the operative side disappeared in 75% of the patients, with enlarged cerebral peduncle on the healthy side.

**Figure 4:**
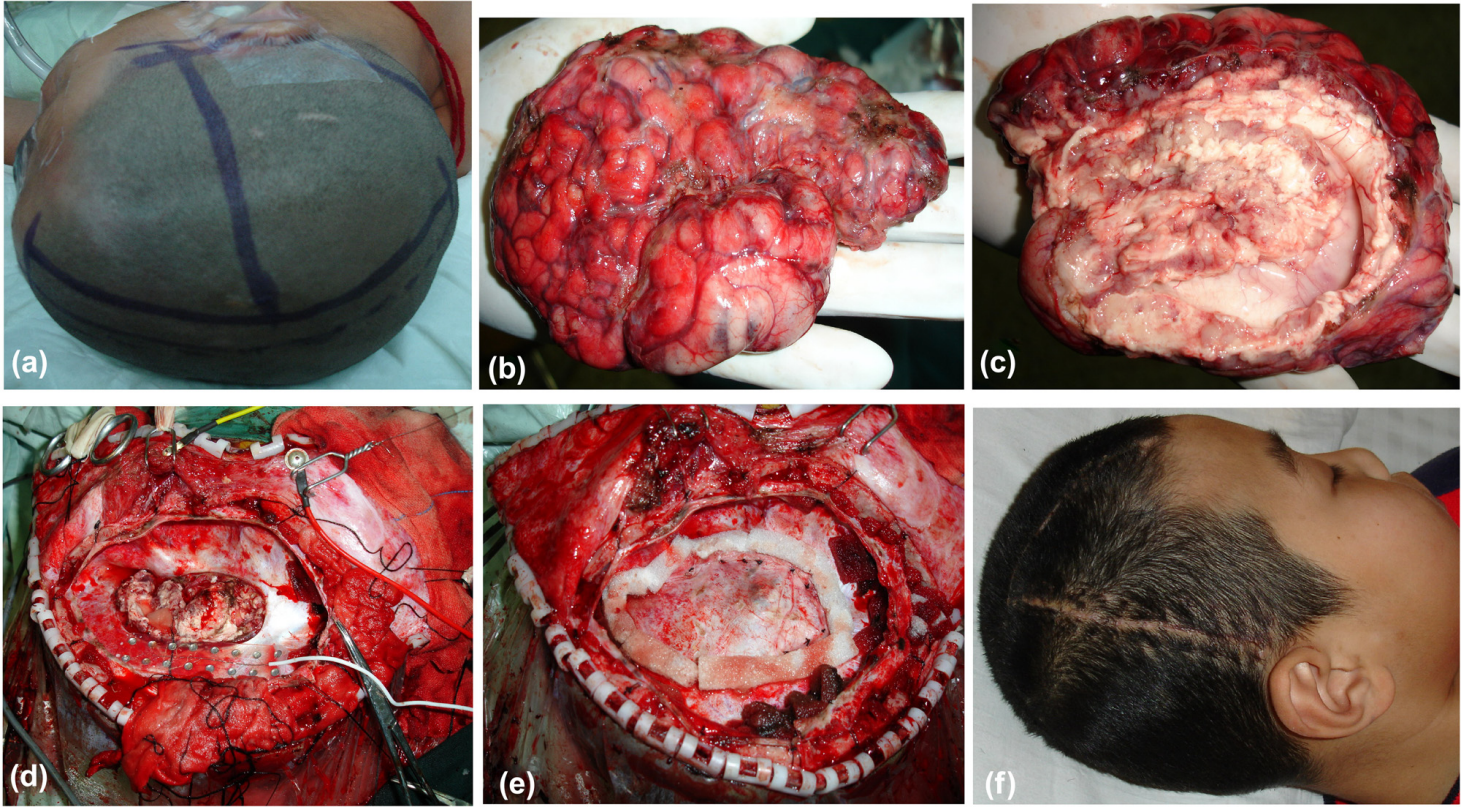
This patient is the same as in Figure 2. (a) The head was marked for operation. (b) The convex surface of the excised cerebral hemisphere was shown. (c) The internal surface of the excised cerebral hemisphere was shown. (d) The basal ganglia and thalamus were preserved after hemispherectomy. (e) The dura mater was folded and sutured to reduce the subdural space on the operative side. (f) Follow-up 3 years later.

**Figure 5:**
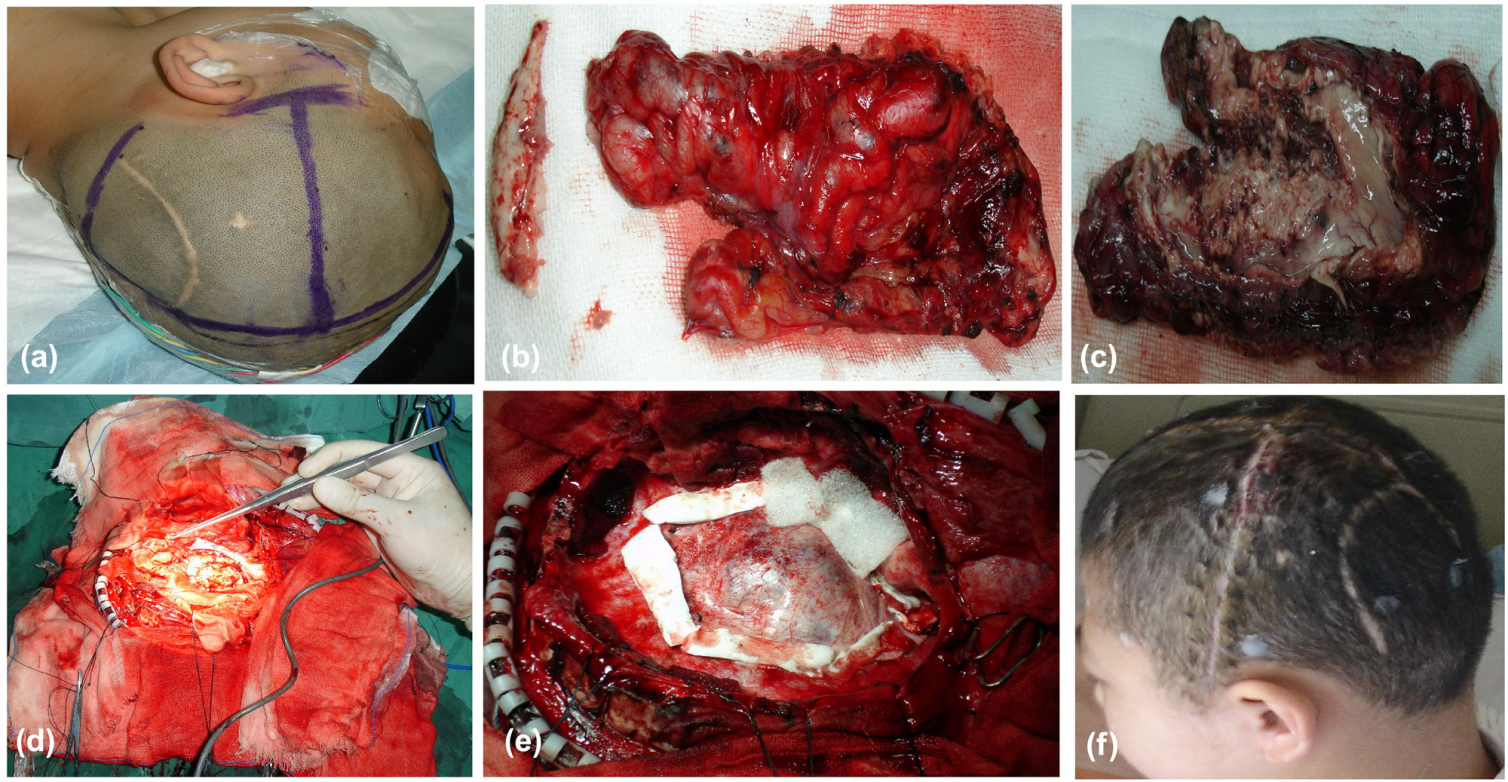
A 9-year-old boy who had medically intractable epilepsy was treated with anatomical hemispherectomy. (a) The head was marked for operation. (b) The convex surface of the excised cerebral hemisphere was shown. (c) The internal surface of the excised cerebral hemisphere was shown. (d) The basal ganglia and thalamus were preserved after hemispherectomy. (e) The dura mater was folded and closed with suturing to reduce the subdural space on the operative side. (f) Follow-up 2 years later.

According to the EEG, the basilic rhythm was primarily *α* and *β* waves, the spike waves disappeared, the wave amplitude apparently decreased, and physiological waves increased with time (Figures 4 and 5).

## 5 Discussion

In this study, we investigated the effect of modified hemispherectomy on patients with infantile hemiparesis and medically intractable epilepsy and changes in the brain and skull on medical imaging. The modified hemispherectomy results in satisfactory control of epilepsy with seizure relief in 91% (*n* = 39) of patients (classified as Engel I) and basic control in 9% (*n* = 4, Engel II) at a mean follow-up duration of 11 years. The intracranial space on the operative side significantly shrank and the healthy cerebral hemisphere shifted toward the lesioned side, with enlarged lateral ventricle on the healthy side, thickened skull, and enlarged frontal sinus on the lesioned side. After 4–5 years, the intracranial space on the lesioned side disappeared in 75% of the patients, with a greater cerebral peduncle on the healthy side.

In this study, we modified the hemispherectomy at the later stage to handle the residual cavity after the diseased hemisphere was removed. First, an appropriate slice of the muscle was used to occlude the interventricular foramen of Monro and fixed onto the frontal and rare parts of the cerebral falx. The residual space surrounding the muscle slice was then sealed with biological glue so as to prevent bloody fluid in the residual cavity from entering the healthy side. Second, the convexal dura mater on the hemispherectomy side was completely cut out, made into a piece of an appropriate size, and sutured onto the cerebellar tentorium for complete sealing of the cerebellar tentorial foramen before reinforcement with biological glue to seal the surrounding residual gap. This step decreased the subdural space to the greatest extent and completely cutoff the link between the subdual and epidural spaces. This approach was different from the traditional way, which was to overturn and suture the convexal dura mater onto the cerebellar tentorium for sealing the cerebellar tentorial foramen without reinforcement of the sealing. Moreover, the piece of dura mater which was sutured onto the cerebellar tentorium could support and protect the cerebral peduncle and basal ganglia on the hemispherectomy side. After hemispherectomy, bloody cerebrospinal fluid was drained for 3–5 days until the cerebrospinal fluid became clear.

The modified hemispherectomy was to prevent delayed complications especially the superficial hemosiderosis of the brain and spinal cord [1,14]. This is the most severe complication and may occur many years after hemispherectomy, with an incidence of 18–35% [22]. In patients with hemispherectomy, about 30% of patients experienced this complication 4 years after the procedure, and this complication may occur 4–37 years after hemispherectomy, with a mean time of 8 years [2,14,23]. The pathophysiology of the superficial cerebral hemosiderosis following traditional anatomical hemispherectomy involves the formation of hemorrhagic membrane in the hemispherectomy space, resulting in chronic hemorrhage and siderosis of the ependyma and subarachnoid surfaces [24]. Blocking of the aqueduct of the Sylvius may cause hydrocephalus [23]. The clinical presentation of this complication is recurrent headache, cerebellar ataxia, neurological deafness, obstructive hydrocephalus, and progressive mental retardation, leading to a mortality rate of 25–50%. Our modified hemispherectomy had completely blocked the communication of bloody cerebrospinal fluid between the contralateral and the hemispherectomy sides, so that no blood or blood products entered the healthy side to cause the superficial hemosiderosis. When followed up for 7–15 years (mean 11.3), no patients had this complication.

The modified hemispherectomy also showed good effects on epilepsy control, resulting in Engel I in 91% and Engel II in 9% at follow-up of 7–15 years. In other reports, the rate of seizure freedom following hemispherectomy has been reported to be 52–89%, with a link in improved seizure control and cognitive motor outcomes [3,9–11]. After hemispherectomy, the abnormal waves on EEG on the healthy hemisphere might disappear completely or greatly reduce in the frequency clinically. In patients with longer history of the disease, frequent epilepsy might cause an epileptogenic lesion on the healthy hemisphere, and some independent epileptogenic lesions on the healthy side may suggest continued application of antiepileptics, which may be the primary reason for some patients who did not have good epilepsy control. At long-term follow-up, the healthy hemisphere shifted toward the hemispherectomy side, and the EEG waves on the hemispherectomy side were lower than that on the healthy side. The epileptiform discharge on the healthy side was also greatly decreased in the frequency compared with that before the surgery or 1 month after the surgery. This may suggest that hemispherectomy should be performed as early as possible because abnormal discharge in the diseased hemisphere may continuously affect the development and normal function of the healthy hemisphere [25]. Great intensity of seizure activities has been linked with mental status, whereas early surgery can cause better development outcomes [26]. Early hemispherectomy for epilepsy helps avoiding anticonvulsant exposure during critical stages of brain development, and exposure to anticonvulsant drugs in infancy is associated with psychiatric and neurological sequelae in humans and neurological apoptosis in animals [27,28].

Based on posthemispherectomy medical imaging, it was observed that the operative space shrank, and the midline structures shifted markedly toward the operative cavity, with the expanded lateral ventricle on the healthy side and the thickened skull and enlarged frontal sinus on the operative side. The intracranial space on the operative side disappeared in 75% of the patients in 4–5 years, with enlarged cerebral peduncle on the healthy side. Marked shift in the midline structures of the brain indicates successful obliteration of the foramen of Monro. If the foramen was patent, the pressure on both the healthy and operative sides would be equivalent, and the development of hydrocephalus would have prevented the midline structures from shifting toward the operative side. The ventricular cerebrospinal fluid was drained in hemispherectomy space until the fluid was clear and colorless, which implied no perfusion of blood from the operative space. Moreover, the cerebrospinal fluid circulation is unobstructed; and if there is no absorption barrier, there will be no hydrocephalus. Following hemispherectomy, fundoscopy showed the fundus artery to vein ratio of ≥2:3 and a clear boundary of the optic disc. These signs indicated no enlargement of the fundus vein, unobstruction of venous return, and consequently no hydrocephalus.

In the hemispherectomy, the whole diseased cerebral hemisphere was anatomically resected, and the choroid plexus secreting cerebrospinal fluid was also been resected. Only the remaining basal ganglia and thalamus were reserved. The interventricular foramen was sealed with free muscle tissue and glue. The subdural space was closed by the folded dura and glue. The epidural space was injected with normal saline and placed with epidural drainage tube. In these circumstances, the intracranial pressure should not increase. With absorption of the epidural saline and exudative bloody fluid, the intracranial pressure on the operation side is reduced, which makes the structure of the contralateral side gradually to move to the operation side. In addition, with the thickening of skull and dura, the “acceptable” expansion of the contralateral ventricle slowly and gradually fills the space of the brain tissue on the resection side.

In our study, five patients had decreased muscle strength when compared with that before hemispherectomy; however, the muscle strength was quickly resumed. After hemispherectomy, no changes occurred in the muscle strength in the other 38 cases. In the long run, the muscle strength in all patients markedly increased. Due to an injury in the hemisphere, the healthy hemisphere demonstrated compensatory growth to gradually substitute for some functions implemented previously by the injured hemisphere, which can be shown by the asymmetry of bilateral cerebral peduncles. Before and after the surgery, medical imaging revealed greater cerebral peduncle on the healthy side than on the hemispherectomy side, indicating atrophic cerebral peduncle on the hemispherectomy side but compensatory enlargement of cerebral peduncle on the healthy side. This is the anatomical basis for nondeteriorating hemiparesis after hemispherectomy. The changes in the asymmetry of bilateral cerebral peduncles are ascribed to structural and functional reorganization and plasticity; and following hemispherectomy, the nerve fibers dominating motor and sensory functions from the affected hemisphere ipsilateral to the atrophic cerebral peduncle decrease in number and function probably because of Wallerian degeneration [29–31]. In children, the motor pathways are different from those in adults. The nonintersecting corticospinal tract on the intact hemisphere is the anatomical basis of motor functional recovery in patients with early hemispherectomy [32–34]. The nonintersecting corticospinal tract on the intact hemisphere controls the ipsilateral limbs, and these compensatory nerve fibers grow larger and pass through the ipsilateral cerebral peduncle which has consequently compensatory enlargement in children with infantile hemiparesis. If the cerebral peduncle was found to be atrophic or thin in the affected hemisphere compared with that on the healthy side, it can be understood that the function of the affected hemisphere has been replaced by the healthy hemisphere and the motor function not affected by hemispherectomy. After hemispherectomy, the corresponding limb spasm would apparently improve. Muscle strength will be resumed after proper rehabilitation.

In these patients with infantile hemiparesis and intractable epilepsy, epileptic mental disorder was usually reported, causing abnormal personality and behavior of impulse, aggression, and irritability. After hemispherectomy, a relief in the epileptic mental disorder was observed, which may be related to changes in intrabrain structures, resulting in improved number and distribution of the psychoactive transmitters. The CGAS score used to evaluate the social, behavioral, and emotional functioning of the patients significantly improved at follow-up. Further research is necessary to elucidate the specific mechanism.

In this study, we performed anatomical hemispherectomy [15]. Anatomical hemispherectomy preserves only the basal ganglia and thalamus on the operative side. The earliest hemispherectomy did not close the interventricular foramen and did not shrink the subdural space on the operative side, which made extensive wound on the operative side open to the contralateral cerebrospinal fluid circulation. This fashion of procedure provided the basis for the two major complications: hydrocephalus and hemosiderin. In 1983, Adams revised the anatomic hemispherectomy by plugging the Monro foramen with muscle and plicating the convexal dura to the cerebral falx and the cerebellar tentorium at the middle cranial fossa [15]. In our modified anatomical hemispherectomy, the muscle plug was sutured to the cerebral falx and cerebellar tentorium, and the space surrounding the muscle piece was tightly sealed with biological glue. Moreover, when plicating and suturing the convexal dura to the cerebral falx and the cerebellar tentorium, the subdural space was made as small as possible, and the suture sites were glued tightly for complete seal to prevent bloody fluid in the epidural space from entering the subdural space on the hemispherectomy side.

Functional hemispherectomy is a disconnection procedure for medically severe refractory epilepsy in which the seizure foci are diffusely locate in one hemisphere [35]. This procedure was first performed by Rasmussen in 1974 and is an improvement of anatomical hemispherectomy. Less invasive surgical procedure and refinement have been achieved to improve the seizure freedom and decrease surgical morbidity and complications. In functional hemispherectomy, only the parietal and temporal lobes were resected, the corpus callosum was cut open completely, the frontal lobe and occipital lobe were preserved anatomically, and the corpus callosum and superior brain stem were completely separated. This procedure can reduce the scope of operation, the trauma, and the incidence of complications. However, functional hemispherectomy may not be better than anatomical hemispherectomy in the clinical outcomes, and we may still perform anatomical hemispherectomy in suitable cases because anatomical hemispherectomy greatly improved with significantly decreased complications.

Some limitations in this study, including only Chinese ethnicity, retrospective, and single-center study with no control group, may potentially affect the publication bias. Future studies will have to solve these issues for better outcomes.

In conclusion, further modified hemispherectomy in patients with infantile hemiparesis and medically refractory epilepsy has markedly ameliorated the effects on epilepsy control and prevention of superficial cerebral hemosiderosis in the long-term follow-up.

## References

[1] Bahuleyan B, Robinson S, Nair AR, Sivanandapanicker JL, Cohen AR. Anatomic hemispherectomy: historical perspective. World Neurosurg. 2013;80:396–8. 10.1016/j.wneu.2012.03.020 22480976

[2] Holloway V, Gadian DG, Vargha-Khadem F, Porter DA, Boyd SG, Connelly A. The reorganization of sensorimotor function in children after hemispherectomy. A functional MRI and somatosensory evoked potential study. Brain. 2000;123(Pt 12):2432–44. 10.1093/brain/123.12.2432 11099446

[3] Lin Y, Harris DA, Curry DJ, Lam S. Trends in outcomes, complications, and hospitalization costs for hemispherectomy in the united states for the years 2000–2009. Epilepsia. 2015;56:139–46. 10.1111/epi.12869 25530220

[4] Baumgartner JE, Blount JP, Blauwblomme T, Chandra PS. Technical descriptions of four hemispherectomy approaches: from the pediatric epilepsy surgery meeting at gothenburg 2014. Epilepsia. 2017;58(Suppl 1):46–55. 10.1111/epi.13679 28386922

[5] Krynauw RW. Infantile hemiplegia treated by removal of one cerebral hemisphere. S Afr Med J. 1950;24:539–46. 15442596

[6] Cook SW, Nguyen ST, Hu B, Yudovin S, Shields WD, Vinters HV, et al. Cerebral hemispherectomy in pediatric patients with epilepsy: comparison of three techniques by pathological substrate in 115 patients. J Neurosurg. 2004;100:125–41. 10.3171/ped.2004.100.2.0125 14758940

[7] Rasmussen T. Hemispherectomy for seizures revisited. Can J Neurol Sci. 1983;10:71–78. 10.1017/s0317167100044668 6861011

[8] Schramm J, Behrens E, Entzian W. Hemispherical deafferentation: an alternative to functional hemispherectomy. Neurosurgery. 1995;36:509–15; discussion 515–506. 10.1227/00006123-199503000-00010 7753351

[9] Lew SM, Koop JI, Mueller WM, Matthews AE, Mallonee JC. Fifty consecutive hemispherectomies: outcomes, evolution of technique, complications, and lessons learned. Neurosurgery. 2014;74:182–94; discussion 195. 10.1227/NEU.0000000000000241 PMC3916907 24176954

[10] Moosa AN, Gupta A, Jehi L, Marashly A, Cosmo G, Lachhwani D, et al. Longitudinal seizure outcome and prognostic predictors after hemispherectomy in 170 children. Neurology. 2013;80:253–60. 10.1212/WNL.0b013e31827dead9 23223541

[11] Schramm J, Kuczaty S, Sassen R, Elger CE, von Lehe M. Pediatric functional hemispherectomy: outcome in 92 patients. Acta Neurochir (Wien). 2012;154:2017–28. 10.1007/s00701-012-1481-3 22941395

[12] White HH. Cerebral hemispherectomy in the treatment of infantile hemiplegia; review of the literature and report of two cases. Confin Neurol. 1961;21:1–50. 13784824

[13] Vadera S, Moosa AN, Jehi L, Gupta A, Kotagal P, Lachhwani D, et al. Reoperative hemispherectomy for intractable epilepsy: a report of 36 patients. Neurosurgery. 2012;71:388–92; discussion 392–383. 10.1227/NEU.0b013e31825979bb 22513844

[14] Kalkanis SN, Blumenfeld H, Sherman JC, Krebs DE, Irizarry MC, Parker SW, et al. Delayed complications thirty-six years after hemispherectomy: a case report. Epilepsia. 1996;37:758–62. 10.1111/j.1528-1157.1996.tb00648.x 8764815

[15] Adams CB. Hemispherectomy – a modification. J Neurol Neurosurg Psychiatry. 1983;46:617–9. 10.1136/jnnp.46.7.617 PMC1027479 6886697

[16] Peacock WJ, Wehby-Grant MC, Shields WD, Shewmon DA, Chugani HT, Sankar R, et al. Hemispherectomy for intractable seizures in children: a report of 58 cases. Childs Nerv Syst. 1996;12:376–84. 10.1007/BF00395089 8869773

[17] Du XY, Chen SC, Guan YG, Gu JJ, Zhao M, Li TF, et al. Asymmetry of cerebral peduncles for predicting motor function restoration in young patients before hemispherectomy. World Neurosurg. 2018;116:e634–9. 10.1016/j.wneu.2018.05.057 29777895

[18] Mullin JP, Soni P, Lee S, Jehi L, Naduvil Valappi AM, Bingaman W, et al. Volumetric analysis of cerebral peduncles and cerebellar hemispheres for predicting hemiparesis after hemispherectomy. Neurosurgery. 2016;79:499–507. 10.1227/NEU.0000000000001307 27322806

[19] Chan AY, Urgun K, Tran DK, Kyong T, Hsu FPK, Vadera S. Cerebral peduncle volume and motor function following adult hemispherectomy. World Neurosurg. 2019;126:156–9. 10.1016/j.wneu.2019.03.034 30877000

[20] Danielsson S, Viggedal G, Steffenburg S, Rydenhag B, Gillberg C, Olsson I. Psychopathology, psychosocial functioning, and IQ before and after epilepsy surgery in children with drug-resistant epilepsy. Epilepsy Behav. 2009;14:330–7. 10.1016/j.yebeh.2008.10.023 19026763

[21] Schorre BE, Vandvik IH. Global assessment of psychosocial functioning in child and adolescent psychiatry. A review of three unidimensional scales (CGAS, GAF, GAPD). Eur Child Adolesc Psychiatry. 2004;13:273–86. 10.1007/s00787-004-0390-2 15490275

[22] Tinuper P, Andermann F, Villemure JG, Rasmussen TB, Quesney LF. Functional hemispherectomy for treatment of epilepsy associated with hemiplegia: rationale, indications, results, and comparison with callosotomy. Ann Neurol. 1988;24:27–34. 10.1002/ana.410240107 3137858

[23] O’Brien DF, Basu S, Williams DH, May PL. Anatomical hemispherectomy for intractable seizures: excellent seizure control, low morbidity and no superficial cerebral haemosiderosis. Childs Nerv Syst. 2006;22:489–98; discussion 499. 10.1007/s00381-005-0023-1 16470390

[24] Hughes JT, Oppenheimer DR. Superficial siderosis of the central nervous system. A report on nine cases with autopsy. Acta Neuropathol. 1969;13:56–74. 10.1007/BF00686141 5784099

[25] Gonzalez-Martinez JA, Gupta A, Kotagal P, Lachhwani D, Wyllie E, Luders HO, et al. Hemispherectomy for catastrophic epilepsy in infants. Epilepsia. 2005;46:1518–25. 10.1111/j.1528-1167.2005.53704.x 16146448

[26] Kumar RM, Koh S, Knupp K, Handler MH, O’Neill BR. Surgery for infants with catastrophic epilepsy: An analysis of complications and efficacy. Childs Nerv Syst. 2015;31:1479–91. 10.1007/s00381-015-2759-6 26022500

[27] Gedzelman ER, Meador KJ. Neurological and psychiatric sequelae of developmental exposure to antiepileptic drugs. Front Neurol. 2012;3:182. 10.3389/fneur.2012.00182 PMC3530733 23293628

[28] Meador KJ, Loring DW. Risks of in utero exposure to valproate. JAMA. 2013;309:1730–1. 10.1001/jama.2013.4001 PMC3685023 23613078

[29] Graveline CJ, Mikulis DJ, Crawley AP, Hwang PA. Regionalized sensorimotor plasticity after hemispherectomy fMRI evaluation. Pediatr Neurol. 1998;19:337–42. 10.1016/s0887-8994(98)00082-4 9880136

[30] Wakamoto H, Eluvathingal TJ, Makki M, Juhasz C, Chugani HT. Diffusion tensor imaging of the corticospinal tract following cerebral hemispherectomy. J Child Neurol. 2006;21:566–71. 10.1177/08830738060210071401 16970845

[31] Zsoter A, Pieper T, Kudernatsch M, Staudt M. Predicting hand function after hemispherotomy: TMS versus fmri in hemispheric polymicrogyria. Epilepsia. 2012;53:e98–101. 10.1111/j.1528-1167.2012.03452.x 22462681

[32] Benecke R, Meyer BU, Freund HJ. Reorganisation of descending motor pathways in patients after hemispherectomy and severe hemispheric lesions demonstrated by magnetic brain stimulation. Exp Brain Res. 1991;83:419–26. 10.1007/BF00231167 2022247

[33] Jang SH, Byun WM, Chang Y, Han BS, Ahn SH. Combined functional magnetic resonance imaging and transcranial magnetic stimulation evidence of ipsilateral motor pathway with congenital brain disorder: A case report. Arch Phys Med Rehabil. 2001;82:1733–6. 10.1053/apmr.2001.25101 11733891

[34] Nirkko AC, Rosler KM, Ozdoba C, Heid O, Schroth G, Hess CW. Human cortical plasticity: functional recovery with mirror movements. Neurology. 1997;48:1090–3. 10.1212/wnl.48.4.1090 9109906

[35] Young CC, Williams JR, Feroze AH, McGrath M, Ravanpay AC, Ellenbogen RG, et al. Pediatric functional hemispherectomy: operative techniques and complication avoidance. Neurosurg Focus. 2020;48:E9. 10.3171/2020.1.FOCUS19889 32234987

